# Quantization of nonequilibrium heat transport models based on isomorphism and gauge symmetry

**DOI:** 10.1038/s41598-025-93640-y

**Published:** 2025-04-28

**Authors:** Chen Yang

**Affiliations:** Faculty of Science and Engineering, The University of Manchester, Manchester, M13 9PL UK

**Keywords:** Physics, Quantum physics, Theoretical physics

## Abstract

The diffusive model in a local thermal equilibrium medium has been well established for classical heat transport. In this study, we investigated the gauge potential formulation of a heat transfer model in a non equilibrium system within classical and quantum frameworks. To achieve this, scalar and vector potential and gauge functions were first introduced to characterize the heat transport model. Subsequently, minimal coupling of the heat potential was established via isomorphic mapping between the heat transport and electromagnetism. The Schrödinger equation with quantized heat potentials that fulfill the gauge symmetry is established. Based upon, we further studied the quantization of enthalpy and entropy from a reversible thermodynamic process, including continuous and discretized system. Later, the connections between the non-isentropic condition and gauge symmetry violation were revealed to categorize classical-permitted and quantum-permitted processes. To support the study, thermal quantities are calculated according to the recent report in literature for the two predicted heat transport modes. Theoretically, it has been shown that the quantization of heat potentials as a consequence of isomorphic characterization and gauge symmetry. By incorporating the critical temperature and local symmetry breaking, it interprets the transition of quantum formulation to classical formulation in finite spatial and temporal limits.

## Introduction

Historically, the transportation of heat in bulk materials has been formulated as a diffusive-like process governed by Fourier’s law in a local thermal equilibrium state^1^. Local thermal equilibrium extends the concept of global thermal equilibrium to allow a temperature gradient owing to the natural heat-transport mechanism^2,3^. During the last few decades, continuous research and studies on second sounds and heat waves in different materials (e.g.^3^He–^4^He, NaF, NaI, and sapphire) have revealed the importance of non equilibrium heat transport mechanisms that differ from diffusive-like processes in the quantum regime^4–10^. More recently, experimental studies of novel materials (e.g., SrTiO_3_, graphite, BKT fluid, and Fermi gas) at low temperatures have further enriched the observation and understanding of heat transport in different media through wave-like mechanisms^11–15^. The continuous discovery of heat wave phenomena in microscopic systems has attracted increasing interest in both fundamental physics and quantum technology^16^.

In the past, wave-like heat transportation through the spatial domain was studied from a theoretical perspective before the first measurement of heat waves (e.g., the second sound), which dates back to the work of Maxwell^17^. Since it was originally proposed by Cattaneo in the formulation of the hyperbolic heat-transport equation (also call Maxwell–Cattaneo equation), this heat-transport equation has been studied and extended by several authors^18,19^. Comprehensive reviews of the Maxwell–Cattaneo model have shown many attractive properties, including consistency of local causality and energy conservation in thermodynamics^1^. In parallel with the study in physics, the topic of non-equilibrium heat transport was also investigated in applied mechanics for thermoelastic materials at a small scale^20^. The hyperbolic features of the Maxwell–Cattaneo model have provided new insights when thermal flux is coupled with material elasticity, as in solids and fluids^21,22^. More recently, studies on the Maxwell–Cattaneo model have brought further attention to engineering mechanics and heat transfer^23,24^.

From the literature, the study of the non equilibrium heat transport model is based on the modification of Fourier’s law. Simultaneously, the model can be investigated from the gauge-potential formulation aspect, which shows a direct link to field quantization and classical transition. The purpose of this study was to quantize the heat potentials and study their connections with thermodynamic processes. First, we introduce the gauge function and heat potentials and show the connection between the heat and electromagnetic potentials. Next, we investigated the quantization of heat potentials under the local gauge symmetry of the wave function. Subsequently, we studied the enthalpy and entropy changes of quantum heat potentials for a continuous and discretized thermodynamic system. The violation of the local gauge symmetry of the heat potentials in the decay form is highlighted to demonstrate the connections between the non-isentropic condition and the quantum-to-classical transient process. Finally, by illustrating the isomorphic characterizations of the parallel particle and field models, we highlight the entropy change as a critical viewpoint for categorizing classical permitted and quantum-permitted processes. The non-isentropic process is linked to the exponential decay of the wave function from the initial quantum form to the final classical form.

## Results

### Gauge-potential formulation of heat

We first investigated the gauge-potential formulation of the heat-transport model and demonstrated the role of scalar and vector heat potentials. We then show the analogy gauge and field potentials compared with classical electromagnetism.

#### Potentials of heat transport models

In a conventional heat transport model, the heat equation is usually established using Fourier conduction laws. If the nonlocal heat transport is considered, the modified relation in Eq. (1) was proposed in literature^1^.

1 $${\varvec{q}}=-\kappa \nabla T-{\tau }_{0}{\partial }_{t}{\varvec{q}},$$

where ${\varvec{q}}$ is the (nonlocal) heat flux density, $T$ is the temperature, $\kappa$ is the thermal conductivity, and ${\tau }_{0}$ is the relaxation time, which reflects the material properties. A vector heat potential ${\varvec{F}}$ can be introduced to link its rate over the relaxation time and the heat flux density:

2 $$\frac{{\varvec{F}}}{{\tau }_{0}}=-{\varvec{q}} \Rightarrow {\varvec{F}}=-{\tau }_{0}{\varvec{q}}.$$

Substitution of Eq. (3) into Eq. (2) yields the following relation for the modified heat-flux density:

3 $${\varvec{q}}=-\kappa \nabla T+{\partial }_{t}{\varvec{F}}.$$

According to^1^, the heat equation based on Eq. (3) is in a hyperbolic form:

4 $${\nabla }^{2}T+\frac{1}{\alpha }{\partial }_{t}T-\frac{1}{{{\varvec{c}}}_{h}^{2}}{\partial }_{t}^{2}T={f}_{h}\left({\varvec{r}},t\right) ; {{\varvec{c}}}_{h}=\sqrt{\frac{\kappa }{{\tau }_{0}\rho {C}_{p}}}$$

where ${{\varvec{c}}}_{h}$ denote the phase velocity, $\alpha$ denotes the thermal diffusivity, and where ${f}_{h}$ denotes the source term, respectively. Next, a wave-like gauge function ${\Lambda }_{h}$ of the heat transport model can be introduced to connect the scalar potential (temperature) and vector potential, as follows:

5 $$\nabla \cdot {\varvec{F}}+\frac{1}{{{\varvec{c}}}_{h}^{2}}{\partial }_{t}T=0.$$

and

6 $$\Delta T={T}^{\prime}-T=-{\partial }_{t}{\Lambda }_{h}, \Delta {\varvec{F}}={{\varvec{F}}}^{{\prime}}-{\varvec{F}}=\nabla {\Lambda }_{h},$$

where $\Delta T$ denotes the change in temperature, $\Delta {\varvec{F}}$ denotes the change in the heat flux for different configurations, $\rho$ is the mass density, and ${C}_{p}$ is the specific heat capacity. Here, the temperature is $T$ considered as the scalar potential. By substituting Eq. (6) into Eq. (5), the introduced heat-gauge function is governed by the following wave equation:

7 $${\nabla }^{2}{\Lambda }_{h}-\frac{1}{{{\varvec{c}}}_{h}^{2}}{\partial }_{t}^{2}{\Lambda }_{h}=0.$$

It is worth noting that two types of gauge solutions satisfy the above equation: wave-like and decay forms. The wave-like form can be obtained from the linear superposition of the plain wave solutions as

8 $${\Lambda }_{h}={\overline{\Lambda } }_{h}{e}^{-i\theta \left({\varvec{r}},t\right)}={\overline{\Lambda } }_{h}{e}^{-i({\varvec{k}}{\varvec{r}}-\omega t)},$$

where ${\overline{\Lambda } }_{h}$ denotes the magnitude, ${\varvec{k}}$ denotes the wave vector, $\omega$ denotes the angular frequency, which satisfies the relationship $\omega ={{\varvec{c}}}_{h}{\varvec{k}}$. The decay form can be obtained from the linear superposition of the exponential decay solution asfollows:

9 $${\Lambda }_{h}={\overline{\Lambda } }_{h}{e}^{-\Omega ({\varvec{r}},t)}={\overline{\Lambda } }_{h}{e}^{-(\boldsymbol{\alpha }{\varvec{r}}+\beta t)},$$

where $\boldsymbol{\alpha }$ is the spatial decay constant, $\beta$ is the temporal decay constant and fulfill the relation $\beta ={{\varvec{c}}}_{h}\boldsymbol{\alpha }$. In a later section, the different quantization behaviors between the wave-like gauge and decay gauge of the heat transport model are discussed.

#### Gauge invariance and functional map

The gauge invariance feature of the heat model can be established using the introduced heat potentials. The heat flux ${\varvec{q}}$ and vorticity ${\varvec{\omega}}$ were not altered by the change in heat potentials from the old to the new configurations ($T\to T^{\prime},{\varvec{F}}\to {\varvec{F}}^{\prime}$), as follows:

10 $${\varvec{q}}=-\kappa \nabla {T}^{\prime}+{\partial }_{t}{{\varvec{F}}}^{{\prime}}=-\kappa \nabla T+{\partial }_{t}{\varvec{F}} ;{\varvec{\omega}}=-\nabla \times {{\varvec{F}}}^{{\prime}}=-\nabla \times {\varvec{F}}.$$

In the gauge-potential form, it is not surprising that the definition of the nonlocal heat flux not only satisfies energy conservation (first law of thermodynamics), but also fulfills gauge invariance. The gauge-invariance feature in Eq. (8) shows a clear analogy with the field strengths of various linear waves in electromagnetism. For instance, in electromagnetism, gauge invariance leads to the same strength in the electromagnetic fields. The aforementioned analogies are summarized in the following table. From Table 1, the thermal charge $\rho {C}_{p}$ in the heat transport is identified, which plays a role similar to that of the electrical charge $q$ in the electromagnetism. In heat transport, the thermal charge reflects the material properties of thermal inertia that resists the change in temperature with a given amount of heat (energy).

**Table 1:** Gauges and field potentials of heat transport and electromagnetism models.

Models:	Heat transport	Electromagnetism
Charge	$\rho {C}_{p}$	$q$
Gauge	${\Lambda }_{h}\left({\varvec{r}},t\right)$	$\Lambda \left({\varvec{r}},t\right)$
Field potential	$T,\boldsymbol{ }{\varvec{F}}$	$V,\boldsymbol{ }{\varvec{A}}$
Field strength	${\varvec{q}},\boldsymbol{ }{\varvec{\omega}}$	${\varvec{E}},\boldsymbol{ }{\varvec{B}}$
Governing equation	${\nabla }^{2}{\Lambda }_{h}-\frac{1}{{{\varvec{c}}}_{h}^{2}}{\partial }_{t}^{2}{\Lambda }_{h}=0$	${\nabla }^{2}\Lambda -\frac{1}{{{\varvec{c}}}^{2}}{\partial }_{t}^{2}\Lambda =0$
	$\Delta T=-{\partial }_{t}{\Lambda }_{h} ; \Delta {\varvec{F}}=\nabla {\Lambda }_{h}$	$\Delta V=-{\partial }_{t}\Lambda ; \Delta {\varvec{A}}=\nabla\Lambda ,$

From the gauge potential formulation, analogies between the theoretical characterization of nonlocal heat transport and classical electromagnetism become apparent. Temperature, as a scalar heat potential, has a role similar to that of the diffusive or wave-like heat transport model. However, the vectorial heat potential is not shown in existing diffusive-like or wave-like heat transport models. In the gauge potential formulation of the heat transport model, the vector heat potential is indispensable to form a complete picture of the heat field. From Eqs. (4) and (6), the governing equation of the vector potentials can be deduced by dividing the phase speed ${{\varvec{c}}}_{h}$ from both sides, as follows:

11 $${\nabla }^{2}{\varvec{F}}+\frac{1}{\alpha }{\partial }_{t}{\varvec{F}}-\frac{1}{{{\varvec{c}}}_{h}^{2}}{\partial }_{t}^{2}{\varvec{F}}={{\varvec{g}}}_{h}({\varvec{r}},t).$$

where ${{\varvec{g}}}_{h}$ is the source term. From the Eqs. (4) and (11), the scalar and vector potentials of the heat field were decoupled from the governing equations. Furthermore, because the gauge functions in the above table are governed by the wave equations, it is feasible to define a functional mapping ${\mathcal{F}}_{h}$ between the two sets of gauge functions as

12 $$q\Lambda ={\mathcal{F}}_{h}\circ \rho {C}_{p}{\Lambda }_{h}={\mathcal{F}}_{h}(\rho {C}_{p}{\Lambda }_{h}),$$

and the field potentials can be represented as,

13 $$qV={\mathcal{F}}_{h}\circ \rho {C}_{p}T={\mathcal{F}}_{h}\left(\rho {C}_{p}T\right) ;q{\varvec{A}}={\mathcal{F}}_{h}\circ \rho {C}_{p}{\varvec{F}}={\mathcal{F}}_{h}(\rho {C}_{p}{\varvec{F}})$$

where $\Lambda$ is the Lorenz gauge in electromagnetism and ${\mathcal{F}}_{h}$ is the functional mapping of the complex phase function that preserves the differential equations before and after mapping.

### Field quantization and gauge symmetry

Using functional mapping between the field potentials, we investigated the minimal coupling of heat potentials and their quantization via gauge symmetry. Simultaneously, the violation of gauge symmetry for heat potentials in the exponential decay form was also studied.

#### Minimal coupling and Schrödinger equation

The coupling between heat potentials and particle dynamics can be formulated based on the modification of the dynamic variables of free particles with additional potential-dependent terms. For a free particle, the conservation of the Hamiltonian and momentum leads to the governing equation of the action integral $S$ as^25,26^

14 $${\nabla }^{2}S-\frac{1}{{{\varvec{v}}}^{2}}{\partial }_{t}^{2}S=0 \Rightarrow S=i{S}_{0}{\phi }_{0}\left({\varvec{r}},t\right),$$

where ${S}_{0}$ denotes the magnitude of the action, and ${\phi }_{0}$ is the phase function of the free particle. In classical electrodynamics, the Hamiltonian and momentum of a charged particle in a time-dependent electromagnetic field can be defined as

15 $$H\left({\varvec{v}},V\right)=\frac{1}{2}m{{\varvec{v}}}^{2}+qV ; {\varvec{p}}({\varvec{v}},{\varvec{A}})=m{\varvec{v}}-q{\varvec{A}},$$

where ${\varvec{v}}$ is the particle velocity, $H$ the Hamiltonian, ${\varvec{p}}$ the momentum, and $m$ the particle mass. By applying the functional mapping in Eq. (9) into the above equation, the canonical equation becomes:

16 $$H({\varvec{v}},T)=\frac{1}{2}m{{\varvec{v}}}^{2}+{\mathcal{F}}_{h}(\rho {C}_{p}T) ; {\varvec{p}}({\varvec{v}},{\varvec{F}})=m{\varvec{v}}-{\mathcal{F}}_{h}(\rho {C}_{p}{\varvec{F}}),$$

Since the Hamiltonian and momentum are governed by action $S$ and its first-order differentiations, the action in Eq. (11) gives the linear operator forms of the dynamic variables and the above equation becomes:

17 $$i{S}_{0}{\partial }_{t}\phi =i{S}_{0}{\partial }_{t}{\phi }_{0}+{\mathcal{F}}_{h}\left(\rho {C}_{p}T\right) ; -i{S}_{0}\nabla \phi =-i{S}_{0}\nabla {\phi }_{0}-{\mathcal{F}}_{h}\left(\rho {C}_{p}{\varvec{F}}\right).$$

where $\phi$ denotes the phase function of the particle coupled with the field potentials. By substituting the above equation into Eq. (13), substituting the action magnitude by the Planck constant ${S}_{0}\to \hslash$ and replacing the phase function with the wave function $\phi \to \psi$, the Schrödinger equation of a particle with heat potentials can be obtained as follows:

18 $$i\hslash {\partial }_{t}\psi =\left(\frac{1}{2m}{\left[-i\hslash \nabla -{\mathcal{F}}_{h}\left(\rho {C}_{p}{\varvec{F}}\right)\right]}^{2}+{\mathcal{F}}_{h}\left(\rho {C}_{p}T\right)\right)\psi .$$

The above equation provides a quantum description of particles coupled with scalar and vector heat potentials, where the heat potentials are given in classical form.

#### Gauge symmetry and quantized heat potentials

To arrive at a quantum description of the scalar and vector heat potentials, we investigated the gauge symmetry of the (quantum) wave function using the gauge function in Eq. (8). In electromagnetism, the Lorenz gauge allows for the invariance of the Schrödinger equation for the wave function in the original and new configurations ($\psi \to \psi ^{\prime}$), as in Eq. (68). For the heat phase function ${\lambda }_{h}$, the invariance of the Schrödinger equation under the gauge transform can be expressed as

19 $${\psi }^{\prime}=\lambda \circ \psi ={e}^{-i{\theta }_{\lambda }\left({\varvec{r}},t\right)}\psi ; \lambda =\lambda (\Lambda )$$

and by applying Eq. (12), we obtain the following relationship from the electromagnetism in Eq. (67),

20 $$\begin{aligned} \frac{\hslash }{q}\lambda \left({\varvec{r}},t\right)&=\Lambda \left({\varvec{r}},t\right)={\mathcal{F}}_{h}\left(\rho {C}_{p}{\Lambda }_{h}\right)\\&=\frac{\hslash }{q}\rho {C}_{p}{\mathcal{F}}_{h}\left({\Lambda }_{h}\right) \Rightarrow {\rho {C}_{p}\Lambda }_{h}\left({\varvec{r}},t\right)=\hslash {\mathcal{F}}_{h}^{-1}\left({\lambda }_{h}\right),\end{aligned}$$

where $\lambda$ denotes the phase function of electromagnetism and ${\mathcal{F}}_{h}^{-1}$ is the inverse map between the heat and electromagnetic potentials and fulfills the unity condition ${\mathcal{F}}_{h}^\circ {\mathcal{F}}_{h}^{-1}=1$. Applying the above relationship to Eq. (7), the quantum description of the heat potential can be obtained as

21 $$\Delta T=-{\partial }_{t}{\Lambda }_{h}=\frac{\hslash \omega }{\rho {C}_{p}}{\mathcal{F}}_{h}^{-1}\left({\lambda }_{h}\right) ; \Delta {\varvec{F}}=\nabla {\Lambda }_{h}=\frac{\hslash {\varvec{k}}}{\rho {C}_{p}}{\mathcal{F}}_{h}^{-1}({\lambda }_{h}).$$

where $\omega$ and ${\varvec{k}}$ denote the angular frequency and the wavevector of the phase function, respectively. The above relation provides a quantum description of the scalar and vector potentials in a nonlocal heat-transport model. For a given volume, the quantized heat (internal) energy $U$ and heat flux ${\varvec{Q}}$ can be obtained from the above equation by multiplying the thermal charge $\rho {C}_{p}$ on both sides of the equation, as follows:

22 $$U=\iiint \rho {C}_{p}\Delta Td{{\varvec{r}}}^{3}=\iiint \hslash \omega {\mathcal{F}}_{h}^{-1}\left({\lambda }_{h}\right) d{{\varvec{r}}}^{3}$$

and

23 $${\varvec{Q}}=\iiint \Delta {\varvec{q}}d{{\varvec{r}}}^{3}=\frac{1}{{\tau }_{0}}\iiint \rho {C}_{p}\Delta {\varvec{F}}d{{\varvec{r}}}^{3}=\frac{1}{{\tau }_{0}}\iiint \hslash {\varvec{k}}{\mathcal{F}}_{h}^{-1}({\lambda }_{h})d{{\varvec{r}}}^{3}.$$

The above relations connect the classical description with the quantum description of scalar and vector heat potentials. For decay form in Eq. (9) and considering the same gauge transform of the wave function as in Eq. (17), we obtain.

24 $${\psi }^{\prime}={e}^{-{\Omega }_{\lambda }\left({\varvec{r}},t\right)}\psi ,$$

Because the function ${e}^{-{\Omega }_{\lambda }\left({\varvec{r}},t\right)}$ is a real bounded monotonic decrease between $[0, 1]$, the exponential decay of the transformed wave function ${\psi }^{\prime}$ decreases as a damped oscillation.

25 $${\psi }^{\prime}={e}^{-{\Omega }_{\lambda }\left({\varvec{r}},t\right)}\psi \le \psi .$$

Therefore, the gauge function in decay form violates the gauge symmetry (e.g., wave function damped to zero) of the Schrödinger equation. The influence of the damped wave function and its associated classical limit, as well as entropy, are discussed in later sections.

In general, the solution of the heat gauge and potentials is represented by a linear combination of the wave form and decay form:

26 $${\Lambda }_{h}={c}_{1}{\overline{\Lambda } }_{h}{e}^{-i{\theta }_{\lambda }\left(r,t\right)}+{c}_{2}{\overline{\Lambda } }_{h}{e}^{-{\Omega }_{\lambda }\left(r,t\right)}={\overline{\Lambda } }_{h}\left({c}_{1}{e}^{-i\theta \left({\varvec{r}},t\right)}+{c}_{2}{e}^{-{\Omega }_{\lambda }\left(r,t\right)}\right),$$

where ${c}_{1}$ and ${c}_{2}$ are the weight coefficients that fulfill the unity condition ${c}_{1}+{c}_{2}=1$. Considering the above solution for the gauge transform in Eq. (19),

27 $${\psi }^{\prime}=\left({c}_{1}{e}^{-i{\theta }_{\lambda }\left(r,t\right)}+{c}_{2}{e}^{-{\Omega }_{\lambda }\left(r,t\right)}\right)\circ \psi ={c}_{1}{e}^{-i{\theta }_{\lambda }\left(r,t\right)}\psi +{c}_{2}{e}^{-{\Omega }_{\lambda }\left(r,t\right)}\psi .$$

The above equation shows that the gauge symmetry of the wave function is only partially satisfied, and its influence on the entropy change and quantum-classical transient is highlighted in the Discussion section. In the next sections, we further study the quantum relations of the thermodynamic potentials that fulfill gauge symmetry.

### Thermodynamic potentials in quantum form

In this subsection, the classical enthalpy and entropy in thermodynamics are studied from the obtained quantized heat potentials. First, we studied the general relations based on thermodynamic potentials and then provided an example of a one-dimensional heat string to explore the discretized entropy.

#### Quantization of enthalpy and entropy

From the quantized internal energy, the enthalpy of the thermodynamic system can be further constructed in the quantum form. By recalling the quantization of the linear acoustic model, the quantum description of the acoustic potentials can be given as^27^:

28 $$\Delta P=\hslash \omega {\mathcal{F}}_{a}^{-1}\left({\lambda }_{a}\right) ; \Delta \rho {\varvec{u}}=\hslash {\varvec{k}}{\mathcal{F}}_{a}^{-1}\left({\lambda }_{a}\right).$$

where $P$ is the pressure, ${\varvec{u}}$ is the material velocity and ${\mathcal{F}}_{a}^{-1}$ is the inverse functional map of the acoustic and electromagnetic potentials. From the definition of enthalpy, $H$ in classical thermodynamics, the quantum form can be obtained using Eqs. (20) and (23):

29 $$\Delta H=\Delta U+\iiint \Delta Pd{{\varvec{r}}}^{3}=\iiint \hslash {\omega }_{h}{\mathcal{F}}_{h}^{-1}\left({\lambda }_{h}\right) d{{\varvec{r}}}^{3}+\iiint \hslash {\omega }_{a}{\mathcal{F}}_{a}^{-1}\left({\lambda }_{a}\right) d{{\varvec{r}}}^{3}.$$

The above quantized enthalpy relation provides insight into the energy transport mechanism through acoustic (elastic) and heat branches. For the usual reversible process in a closed system, the total entropy change can be expressed in the quotient form between the internal energy change and temperature, as follows:

30 $$\Delta S=\frac{\Delta U}{T}=\frac{1}{T}\left({U}_{2}-{U}_{1}\right)=\frac{1}{T}\left[\iiint \hslash {\omega }_{2}{\mathcal{F}}_{h}^{-1}\left({\lambda }_{h}\right) d{{\varvec{r}}}^{3}-\iiint \hslash {\omega }_{1}{\mathcal{F}}_{h}^{-1}\left({\lambda }_{h}\right) d{{\varvec{r}}}^{3}\right] ,$$

where the internal energy is represented by Eq. (22), where the subscript denotes the old and new states. By taking the magnitude of the above equation and neglecting the (complex) phase function ${\lambda }_{h}$, we obtain the magnitude of the entropy change in the reversible process as:

31 $$\left|\Delta S\right|=\left|\frac{\Delta U}{T}\right|=\frac{1}{T}\left|{U}_{2}-{U}_{1}\right|=\frac{1}{T}\left|\hslash {\omega }_{2}-\hslash {\omega }_{1}\right|=\frac{1}{T}\lceil\hslash \Delta \omega \rceil .$$

The above equations suggest that at a given temperature $T$, the magnitude of the entropy change of a closed system is proportional to the variation in the quantized internal energy between the old and new states. In the previous sections, the entropy change is studied in a continuum system, where the boundary condition does not explicitly influence the general solution of the heat potentials in the entire domain of analysis. This allows for the continuity of the eigenvalues (and associated eigenvectors) in the orthogonal space.

#### Discretized entropy in one-dimensional system

For instance, consider a typical one-dimensional heat potential with length $L$ that is subjected to the following boundary conditions:

32 $$T\left(0,t\right)=T\left(L,t\right)=0 ; {\varvec{F}}\left(0,t\right)={\varvec{F}}\left(L,t\right)=0$$

For the time-harmonic solutions in Eq. (29), the allowable wavevector and angular frequency of the heat potentials areas follows:

33 $${{\varvec{k}}}_{m}=\frac{m\pi }{L} ; {\omega }_{m}={{\varvec{c}}}_{h}{{\varvec{k}}}_{m}={{\varvec{c}}}_{h}\frac{m\pi }{L} .$$

By substituting these results into Eq. (47), the allowable entropy change between adjacent modes becomes.

34 $$\left|\Delta S\right|=\frac{1}{T}\left|\hslash {\omega }_{m+1}-\hslash {\omega }_{m}\right|=\frac{\pi }{T}\hslash {{\varvec{c}}}_{h}\left|\frac{m+1}{L}-\frac{m}{L}\right|=\frac{\pi }{TL}\hslash {{\varvec{c}}}_{h}.$$

Entropy change is related to a macroscopic process that reflects the thermodynamic direction of the system. In the microscopic process, entropy change reflects the disorder status of the particle ensemble. Therefore, the relationship in Eq. (50) connects the macroscopic description in classical form with the microscopic description in quantum form, where the constraint on the discretized energy becomes explicit. To investigate the maximum entropy change in the heat string, the discretized entropy relation is maximized as the mode number goes to infinity, $m\to \infty$, that is,

35 $$\left|\Delta {S}_{max}\right|=\frac{1}{T}\left|\hslash {\omega }_{m+1}-\hslash {\omega }_{1}\right|=\frac{2\pi }{T}\hslash {{\varvec{c}}}_{h}L\left|\frac{m+1}{L}\right|\cong \frac{2\pi }{T}\hslash {{\varvec{c}}}_{h}{{\varvec{k}}}_{\infty }\boldsymbol{ }.$$

The discretization of entropy change becomes increasingly significant as the length of the thermodynamic system becomes relatively small. Together with the temperature degrees of freedom, the discretized internal energy leads to discretized entropy in the T**–**S diagram. The minimum resolution of the diagram is constrained by quantization of the internal energy. It reduces to a continuous form in the classical limit as the length approaches infinity, $L\to \infty$.

### Classical and quantum thermodynamic process

In this section, we first study entropy changes in the heat potentials of the wave and decay forms. The isentropic condition and its approximation were highlighted to differentiate between the classical and quantum processes. Subsequently, entropy changes were studied for parallel fields, including acoustics, elasticity, and electromagnetism for comparison.

#### Time-averaged isentropic process

From the gauge-potential formulation of the heat transport model, the heat gauges and potentials can be represented by either a wave form or decay form. First, by considering the wave form in Eq. (11), the time average of the heat vector potential ${{\varvec{F}}}_{avg}$ can be defined asfollows:

36 $${{\varvec{F}}}_{avg}=\frac{1}{{t}_{0}}\int {\varvec{F}}({\varvec{r}},t)dt ; {t}_{0}=\frac{2\pi }{\omega } ,$$

where, ${t}_{0}$ is the wave period. Applying the above relationship to Eq. (2), the time-averaged heat flux can be represented asfollows:

37 $${{\varvec{q}}}_{avg}=-\frac{{\tau }_{0}}{{t}_{0}}\int {\varvec{F}}\left({\varvec{r}},t\right)dt$$

Because the above equation can be rewritten using the heat gauge function in Eq. (7), and using the total (time) differentiation as follows:

38 $${\dot{{\varvec{q}}}}_{avg}=-\frac{{\tau }_{0}}{{t}_{0}}\int \left(\nabla {\dot{\Lambda }}_{h}\right)dt=0 ,$$

where the total differential of the heat gauge ${\dot{\Lambda }}_{h}$ vanishes identically in plane-wave solutions. By substituting Eq. (40) into Eq. (39), the time-averaged heat flux vanishes periodically:

39 $${\dot{{\varvec{q}}}}_{avg}=-\frac{{\tau }_{0}}{{t}_{0}}\int \left(\nabla {\dot{\Lambda }}_{h}\right)dt=0 \Rightarrow {{\varvec{q}}}_{avg}=\int {\dot{{\varvec{q}}}}_{avg}dt+C=-\frac{{\tau }_{0}}{{t}_{0}}\int \left(\nabla {\Lambda }_{h}\right)dt=0 .$$

where integral constant $C$ is assumed to be zero. From the definition of heat transport in Eq. (3) and applying the results in Eqs. (30) and (41), the resultant time-averaged temperature gradient becomes.

40 $$\nabla {T}_{avg}=\frac{1}{\kappa }\left(-{{\varvec{q}}}_{avg}+{\partial }_{t}{{\varvec{F}}}_{avg}\right)=0 \Rightarrow {S}_{avg}=0.$$

The above equation shows that the heat potential in the wave form is associated with the time-averaged isentropic condition, where the total time-averaged heat flux across period ${t}_{0}$ and its integer times vanishes. Similar results have been previously reported^28^. By recalling the previous sections, Eq. (40) suggests that the time-averaged isentropic condition is valid after quantization of the heat potentials based on gauge symmetry in Eq. (19). Therefore, it provides a link between the time-averaged entropy change and quantization of the heat transport model.

The decay form of the heat gauge in Eq. (9), the time average of the heat flux ${{\varvec{q}}}_{avg}$ is defined as follows:

41 $${{\varvec{q}}}_{avg}=-\frac{{\tau }_{0}}{{t}_{0}}\int {\varvec{F}}\left({\varvec{r}},t\right)dt={\overline{\Lambda } }_{h}\boldsymbol{\alpha }\frac{{\tau }_{0}}{{t}_{0}}\int {e}^{-\Omega \left({\varvec{r}},t\right)}dt\ne 0 ,$$

where the last inequality comes from the fact that ${e}^{-\Omega \left({\varvec{r}},t\right)}$ is a monotonic (decreasing) function and deviates from zero over space and time. Therefore, the time average of the heat vector potential is non-vanishing.

42 $${{\varvec{F}}}_{avg}=\frac{1}{{t}_{0}}\int {\varvec{F}}({\varvec{r}},t)dt\ne 0$$

and the resultant time-averaged temperature gradient becomes non-zero as expected.

43 $$\nabla {T}_{avg}=\frac{1}{\kappa }\left(-{{\varvec{q}}}_{avg}+{\partial }_{t}{{\varvec{F}}}_{avg}\right)\ne 0 \Rightarrow {S}_{avg}>0 .$$

From the enthalpy perspective, the above equation reflects the non-zero energy flow in the system, which results in a temperature change based on the material thermal charge $\rho {C}_{p}$. In this classical process, the time evolution of the system is always related to the heat flux, which causes the entropy to increase along a single direction.

#### Isentropic process of linearized waves

In a thermodynamic system, the entropy change can also be studied for the mechanical process in a continuous medium, as well as for the electromagnetic process in a vacuum or dielectric medium. For a continuous medium, the transient work-induced enthalpy of the linear mechanical process can be described by the acoustic and elastic potentials in the fluid and solid media as follows:

44 $$\Delta {H}_{me}\left(P,\sigma ,\tau \right)=\iiint \Delta Pd{{\varvec{r}}}^{3}+\iiint (\Delta \sigma +\Delta \tau )d{{\varvec{r}}}^{3} ,$$

where $\sigma$ denotes the tensile (compression) stress and $\tau$ denotes the shear stress in the solid medium. For the electromagnetic process in a dielectric medium, this transient work-induced enthalpy can be represented by the coupling between the electromagnetic potentials and dynamic variables, as follows:

45 $$\Delta {H}_{em}\left(V,{\varvec{A}}\right)=\frac{1}{2m}\left({\varvec{p}}-q{\varvec{A}}\right)+qV.$$

The total enthalpy of continuous and dielectric medium can be obtained as,

46 $$\Delta {H}_{total}(T,S,P,\sigma ,\tau ,V,{\varvec{A}})=U\left(T,S\right)+\Delta {H}_{me}\left(P,\sigma ,\tau \right)+\Delta {H}_{em}\left(V,{\varvec{A}}\right)$$

and the follow terms in the external work can be obtained as,

47 $$\begin{aligned} W\left(P,\sigma ,\tau ,{\varvec{p}},V,{\varvec{A}}\right) & =\Delta {H}_{total}-U(T,S) \\ &=\iiint \left[\Delta P+\Delta \sigma +\Delta \tau \right]d{{\varvec{r}}}^{3}\\&\quad+\frac{1}{2m}{\left[{\varvec{p}}-{\mathcal{F}}_{a}\left(\rho {\varvec{v}}\right)-{\mathcal{F}}_{l}\left(\rho {\varvec{u}}\right)-{\mathcal{F}}_{t}\left(\rho {\varvec{w}}\right)-q{\varvec{A}}\right]}^{2}+q\Delta V ,\end{aligned}$$

where $W$ denotes the total external work of the system. Moreover, the classical results in Eq. (46) can be further represented in quantum form as follows^27^:

48 $$\begin{aligned}W\left(P,\sigma ,\tau ,\widehat{{\varvec{p}}},V,{\varvec{A}}\right) &=\Delta {H}_{total}-U\left(T,S\right)\\ &=\iiint \left[\hslash {\omega }_{a}{\mathcal{F}}_{a}^{-1}\left({\lambda }_{a}\right)+\hslash {\omega }_{l}{\mathcal{F}}_{l}^{-1}\left({\lambda }_{l}\right)+\hslash {\omega }_{t}{\mathcal{F}}_{t}^{-1}\left({\lambda }_{t}\right)\right]d{{\varvec{r}}}^{3}\\&\quad+\left(\frac{1}{2m}{\left[-i\hslash \nabla -{\mathcal{F}}_{a}\left(\rho {\varvec{v}}\right)-{\mathcal{F}}_{l}\left(\rho {\varvec{u}}\right)-{\mathcal{F}}_{t}\left(\rho {\varvec{w}}\right)-q{\varvec{A}}\right]}^{2}+q\Delta V\right)\psi ,\end{aligned}$$

where ${\lambda }_{l}$ and ${\lambda }_{t}$ are the phase functions of the longitudinal and transverse elastic fields, respectively. From the expression in Eqs. (46) and (47), the external work does not explicitly depend on the temperature or heat-flux terms. Consequently, the thermodynamic system remained isentropic under external work from linear acoustic, elastic, and electromagnetic waves, as listed in the following table. Nevertheless, the isentropic condition of heat transport is violated for entropy changes under arbitrary conditions, and is only fulfilled in the time-averaged condition. Table 2 lists the entropy changes and the associated field variables. A comparison of entropy variations in different field models revealed that the entropy condition differentiates the field potentials of other parallel models (e.g., arbitrary isentropic) from the heat potentials (e.g., time-averaged isentropic and non-isentropic).

**Table 2:** Classification of parallel field models in classical and quantum forms by entropy increase.

Field models	Electromagnetism	Acoustics	Elasticity	Heat transport
Variables (Potentials & gauge)	$V,{\varvec{A}},\Lambda$	$P,{\varvec{v}}, {\Lambda }_{a}$	$\sigma ,{\varvec{u}}, {\Lambda }_{l}$; $\tau ,{\varvec{w}}, {\Lambda }_{t}$	$T,{\varvec{F}}, {\Lambda }_{h}$
Enthalpy	$\Delta {H}_{em}\left(V,{\varvec{A}}\right)$	$\Delta {H}_{ac}\left(P,{\varvec{v}}\right)$	$\Delta {H}_{el}\left(\sigma ,{\varvec{u}},\tau ,{\varvec{w}}\right)$	$\Delta H\left(T,S\right)$
Entropy (Arbitrary)	$S=0$	$S=0$	$S=0$	$S>0$
Entropy (Time-averaged)	${S}_{avg}=0$	${S}_{avg}=0$	${S}_{avg}=0$	${S}_{avg}>0$
				$${S}_{avg}=0$$

### Connections with experimental results

The connections between the present theory and experimental information from the literature are presented in this section. We also include a calculation case from the measurement reported to show presence of wave and decay modes from real system.

#### Wave and diffusive heat transport

In the previous section, we observed the coexistence of two independent gauge functions and their associated heat potentials in classical and quantum forms. From the literature, the wave velocity and angular frequency (or wavevector) of the heat potential can be estimated from the harmonic resonance methods, where the wave speed is given by the material properties as^1^:

49 $${{\varvec{c}}}_{h}=\frac{\omega }{{\varvec{k}}}=\frac{2\pi f}{{\varvec{k}}} .$$

According to the literature, the reported speeds of heat waves ${{\varvec{c}}}_{h}$ in Fermi gas are within the range of several millimeters per second with frequency $f$ in the range of several tens of hertz in the nanokelvin^15,29–32^. Additionally, a particle-like picture of the second sound has been reported in the literature based on the measurement of BKT fluid and other cold atom gases^14,33^. For other systems, such as graphite, the reported speed of heat waves can reach up to several kilometers per second with an oscillation period of sub-nanosecond duration in the temperature range of 100–200 K^12,34^. From the measurement and by applying the relations in Eq. (21), the information on the angular frequency and wavevector is associated with the propagation of the heat potentials carrying quantized energy density and quantized momentum flux density in the wave mode that can be calculate as:

50 $$\left|\rho {C}_{p}\Delta T\right|=\hslash \omega \Rightarrow \left|\rho {C}_{p}\right|=\frac{\hslash \omega }{\Delta T};\,\, \left|\rho {C}_{p}\Delta {\varvec{F}}\right|=\hslash {\varvec{k}} \Rightarrow \Delta {\varvec{F}}=\left|\frac{\hslash {\varvec{k}}}{\rho {C}_{p}}\right|.$$

By using the volume integral of the above relations, as in Eqs. (22) and (23), we obtain the quantized internal energy and heat flux that are constituted by the number of propagating particles (e.g., phonons) with associated wave parameters:

51 $$\left|U\right|=\iiint \left|\rho {C}_{p}\Delta T\right|d{r}^{3}\cong n\hslash \omega ;\,\, \left|{\varvec{Q}}\right|=\iiint \left|\rho {C}_{p}\Delta {\varvec{F}}\right|d{r}^{3}\cong n\hslash {\varvec{k}}.$$

where $n\left(r\right)$ denotes the local particle density (number). From energy conservation, the local particle density can be estimated based on the energy of the measured system. The relation in Eq. (51) provides a quantum picture of propagating heat potential waves.

Moreover, it has been reported that the adjustment of environmental temperature alters heat transport behavior. For instance, in the reported Fermi gas or similar systems, the adjustment of the system temperature has shown the vital role of heat transport in terms of wave and decay modes^15,35–37^. Based on the gauge function in Eq. (9), it is suggested that the decay parameter fulfils the following relations:

52 $${{\varvec{c}}}_{h}=\frac{\beta }{\boldsymbol{\alpha }} ;\,\, \beta =\frac{1}{{t}_{0}-{t}_{1}} ;\,\, \boldsymbol{\alpha }=\frac{1}{{{\varvec{r}}}_{0}-{{\varvec{r}}}_{1}}$$

where ${t}_{0}$ and ${t}_{1}$ refer to the time interval and ${{\varvec{r}}}_{0}$ and ${{\varvec{r}}}_{1}$ refer to the spatial interval of wave amplitude decay to ${e}^{-1}$ of the initial amplitude via logarithmic decrement rule. The decay parameters reflect a rate of reduction in the non-equilibrium of the heat field under free evolution. Moreover, the reported observations in the literature indicate that the weight coefficients of the gauge functions ${\Lambda }_{h}$ should be temperature-dependent, and the Eq. (27) becomes the following:

53 $${\psi }^{\prime}={c}_{1}(T){e}^{-i{\theta }_{\lambda }\left(r,t\right)}\psi +{c}_{2}(T){e}^{-{\Omega }_{\lambda }\left(r,t\right)}\psi$$

and

54 $$0\ll {c}_{1}\left({T}_{c}\ge T\right)<1 ; \,\,{c}_{1}\left({T}_{c}<T\right)\to 0 ,$$

where ${T}_{c}$ denotes the superfluid transition temperature, and $T$ denotes the environment temperature. Equation (54) shows the temperature effect on the weight coefficient of the heat gauges, as well as the heat potentials. Under the condition of gauge symmetry, this behavior has an impact on the classical-permissible and quantum-permissible processes inside the system. For environmental temperatures equal to or lower than the superfluid transition temperature (${T}_{c}\ge T$), the gauge symmetry condition is fulfilled and thus the quantization of heat potentials can be established:

55 $${\psi }^{\prime}={c}_{1}\left(T\right){e}^{-i{\theta }_{\lambda }\left({\varvec{r}},t\right)}\psi .$$

When the environmental temperature is greater than the superfluid transition temperature ($T>{T}_{c}$), the gauge symmetry condition is violated and thus the quantization of heat potentials will not be established:

56 $${\psi }^{\prime}={c}_{2}\left(T\right){e}^{-{\Omega }_{\lambda }\left({\varvec{r}},t\right)}\psi \le \psi .$$

From the experiment and theory, one can obtain a temperature-dependent relation for the heat transport process in a classical-permissible or quantum-permissible regime by combining the superfluid transition temperature (from experiment) and gauge symmetry conditions (from theory).

#### Calculation of thermal quantities from experiment

In this subsection, we apply the presented relations to calculate some of the heat transport properties that link macroscopic heat variables (e.g. heat charge, heat flux, energy and momentum and decay constants) from the wave and decay modes. In a recent study, the measurements of heat waves in low-temperature Fermi gas ensemble have been reported in novel thermography technology^15^. Given the temperature variation $\Delta T$ of the underdamped heat wave (T = 63 nK) in the range of 4 nK, with wave velocity ${{\varvec{c}}}_{h}$ closes to 3.5 mm/s and the frequency of heat waves in the range of 18.1 Hz. The quantized energy density of heat potential waves and quantized momentum density of the heat potential waves can be computed from Eq. (22) and Eq. (23) respectively. The heat charge of the gas can be computed from Eq. (50) in terms of Planck constant, $\left|\rho {C}_{p} \right|=\hslash \omega /\Delta T\cong 2.9{e}^{-24}\, \text{J}/\text{K}{\text{m}}^{3}$. Subsequently, the associated heat flux can be calculated in Eq. (50) $\Delta {\varvec{F}}=\hslash {\varvec{k}}/\rho {C}_{p}\cong 1.1{e}^{-6}\, \text{Ks}/\text{m}$ where the wavevector ${\varvec{k}}$ can be determined from the Eq. (49). Table 3 summarizes the heat transport quantities from the calculation based on the input from experiment (grey).

**Table 3:** Calculation of heat transport quantities based on experimental measurement.

Transport mode	List of quantities		Magnitude	Unit (SI)
Wave modes (T = 63 nK)	Angular frequency	$\omega$	1.13E+02	Rad/s
	Wave velocity	${{\varvec{c}}}_{h}$	3.57E−03	m/s
	Temperature	$\Delta T$	4.00E−09	K
	Wave vector	${\varvec{k}}$	3.18E+04	1/m
	Heat energy (quantum)	$\hslash \omega$	1.19E−32	J
	Heat momentum (quantum)	$\hslash {\varvec{k}}$	2.11E−29	J*s/m
	Heat charge	$\rho {C}_{p}$	2.99E−24	J/(K*m^3^)
	Heat potential vector	$\Delta {\varvec{F}}$	1.12E−06	K*s/m
Decay modes (T = 83 nK)	Logarithmic decrement period	${t}_{log}$	0.025	s
	Temporal decay constant	$\beta$	2.51E+02	Rad/s
	Spatial decay constant	$\boldsymbol{\alpha }$	7.04E+04	1/m
Decay modes (T = 101 nK)	Logarithmic decrement period	${t}_{log}$	0.034	s
	Temporal decay constant	$\beta$	1.84E+02	Rad/s
	Spatial decay constant	$\boldsymbol{\alpha }$	5.17E+04	1/m

In the same study, by adjusting the environmental temperature (T = 83 nK) of the ensemble closes to the critical temperature, the heat transport mode of the Fermi gas is mainly in the diffusive mode. From the time response curve of heat pulse, the inverse of temporal decay constant ${\beta }^{-1}$ is approximately 25 ms. By assuming the similar heat transport velocity ${{\varvec{c}}}_{h}$ in previous condition, the calculated spatial decay constant is given by $\boldsymbol{\alpha }=\beta {{\varvec{c}}}_{h}=7.0{e}^{4} \,1/\text{m}$. By further adjusting the environmental temperature (T = 101 nK) of the ensemble beyond the critical temperature, the heat transport mode of the Fermi gas is dominated by the diffusive mode. From the time response curve of heat pulse, the inverse of temporal decay constant ${\beta }^{-1}$ is approximately 34 ms. The calculated spatial decay constant is given by $\boldsymbol{\alpha }=\beta {{\varvec{c}}}_{h}=5.1{e}^{4}\, 1/\text{m}$. With the decay constants, the normalized exponential curve of heat transport can be determined. As the reported temperature amplitudes are only in nanokelvin range, the associated energy and momentum in the heat waves are much less than the first sound (acoustics). From the calculations, it shows the emerged wave mode or decay modes from Eqs. (8) and (9) in gauge-potential formulations are measurable from experiment at different environment temperatures.

## Discussion

**Fig. 1:**
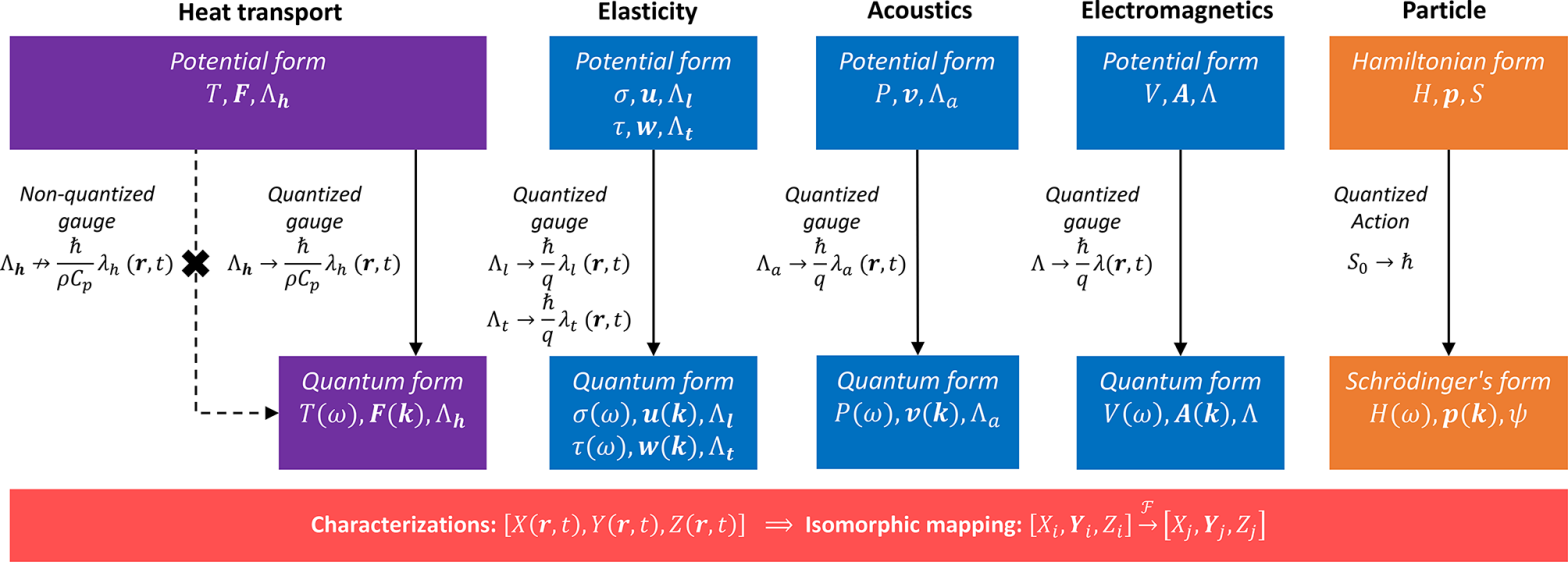
Theoretical characterizations of classical or quantum models from isomorphism aspect.

Firstly, the presence of wave-like and diffusive-like pictures of heat transport in reported experimental studies are investigated from the gauge potential formulation. The two types of gauge functions and their associated scalar and vector heat potentials arise naturally in the non-equilibrium heat transport model. A theoretical diagram is given in Fig. 1 to shows the characterizations of the parallel models via isomorphic physical variables $[X,{\varvec{Y}},Z]$ over space and time. From an algebraic perspective, the above models are similar to different vector spaces that are characterized by analogous coordinates $[{X}_{j},{{\varvec{Y}}}_{i},{Z}_{i}]\to [{X}_{j},{{\varvec{Y}}}_{j},{Z}_{j}]$ under a linear transform^38^. For the wave-like gauge, its quantization of heat potentials can be only achieved under the condition of isomorphic characterization and gauge symmetry. Simultaneously, it was observed that the gauge and heat potentials in the decay form have contributed to an explicit violation of the gauge symmetry. As in the diagram, this feature partially distinguishes the heat-transport model from the other field models.

**Fig. 2:**
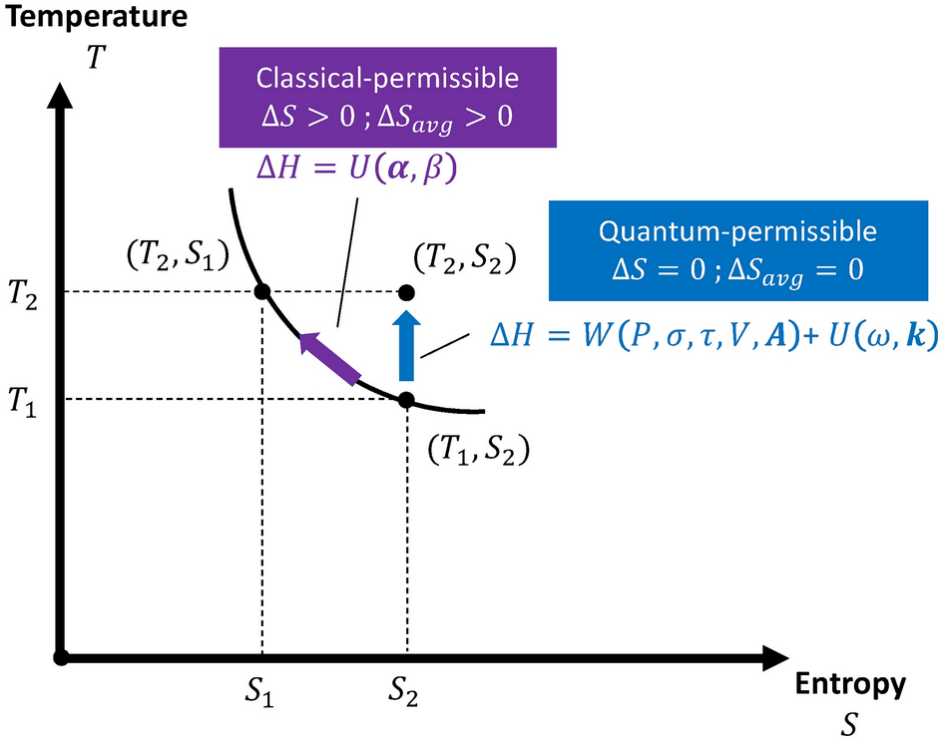
Illustration of the classical-permitted or quantum-permitted process in Temperature–Entropy (T–S) diagram.

Secondly, by combining information of the violation of gauge symmetry and non-isentropic conditions, a direct link between the entropy increases and permissible quantization is revealed. Figure 2 illustrates this link using the temperature**–**entropy (T**–**S) diagram in thermodynamics. In this figure, we consider two types of thermodynamic processes. The first is related to the isentropic and time-averaged isentropic processes with field variables from electromagnetic, acoustic, elasticity, and heat potentials in wave form. The quantization of these field variables has been shown via the gauge symmetry of the wave function in the literature^28^. The second is related to the entropy increase process, which is related to the non-zero heat flux from the heat potential in the decay form. The gauge symmetry of the wave function is explicitly violated in the exponential decay phase; thus, quantization cannot be established. From the above discussion, it can be seen that the entropy increase in the reversible process provides a critical viewpoint on the classical-permitted and quantum-permitted processes. The quantization of field potentials is achievable only for processes that are either arbitrarily isentropic or time-averaged isentropic.

Thirdly, the temperature condition influences the time evolution and quantization of the heat potentials. From a thermodynamic perspective, a system (containing particle ensembles and fields) can be partially isentropic depending on the environmental temperature $T$ and transition temperature ${T}_{c}$ which define the weight coefficients. For infinitesimal variation from the initial condition, the gauge transform with decay functions becomes an infinitesimal transform, and via series expansion, it can be represented as^39^:

57 $${\left.{\psi }^{\prime}\right|}_{{\varvec{r}}\to 0; t\to 0}={c}_{1}(T){e}^{-i\theta \left({\varvec{r}}, t\right)}\psi +{c}_{2}(T){e}^{-\Omega \left({\varvec{r}},t\right)}\psi \cong {c}_{1}I\psi +{c}_{2}\left[I+\Omega +O\left({\Omega }^{2}\right)\right]\psi =\psi$$

where the first-order and second-order terms of $\Omega$ are small under a small time variation; thus, they are approximately identical, which leaves the wave function unaltered. Nevertheless, with finite time variation, the first-order and second-order terms become significant in the above equation, and their damping effect on the wave function cannot be neglected. Eventually, the magnitude of the wave function exponentially damped at a sufficient duration ($\Delta r>0; \Delta t>0$) in the range of (${T}_{c}<T$).

58 $${\left.{\psi }^{\prime}\right|}_{\Delta r>0; \Delta t>0}={c}_{1}\left(T\right){e}^{-i\theta \left(r, t\right)}\psi +{c}_{2}\left(T\right){e}^{-\Omega \left(r,t\right)}\psi \Rightarrow \left|{\psi }^{\prime}\right|={c}_{1}\left|\psi \right|+{c}_{2}{e}^{-\Omega }\left|\psi \right|\ll \left|\psi \right| .$$

The damped wavefunction leads to a transition from a quantum formulation to a typical classical formulation. Moreover, we can introduce a distance-like quantity ${L}_{q}$ to represent the limit at which the quantum wave function is damped to zero under the given temperature conditions $T$ as follows:

59 $$\left|r\right|=\left|{r}_{j}-{r}_{0}\right|\to {L}_{q}(T) ; \,\,{\psi }^{\prime}\left(r,t\right)={\psi }^{\prime}\left({L}_{q},t\right)\cong 0 \Rightarrow {\left|{\psi }^{\prime}\left({L}_{q},t\right)\right|}^{2}\cong 0 .$$

**Fig. 3:**
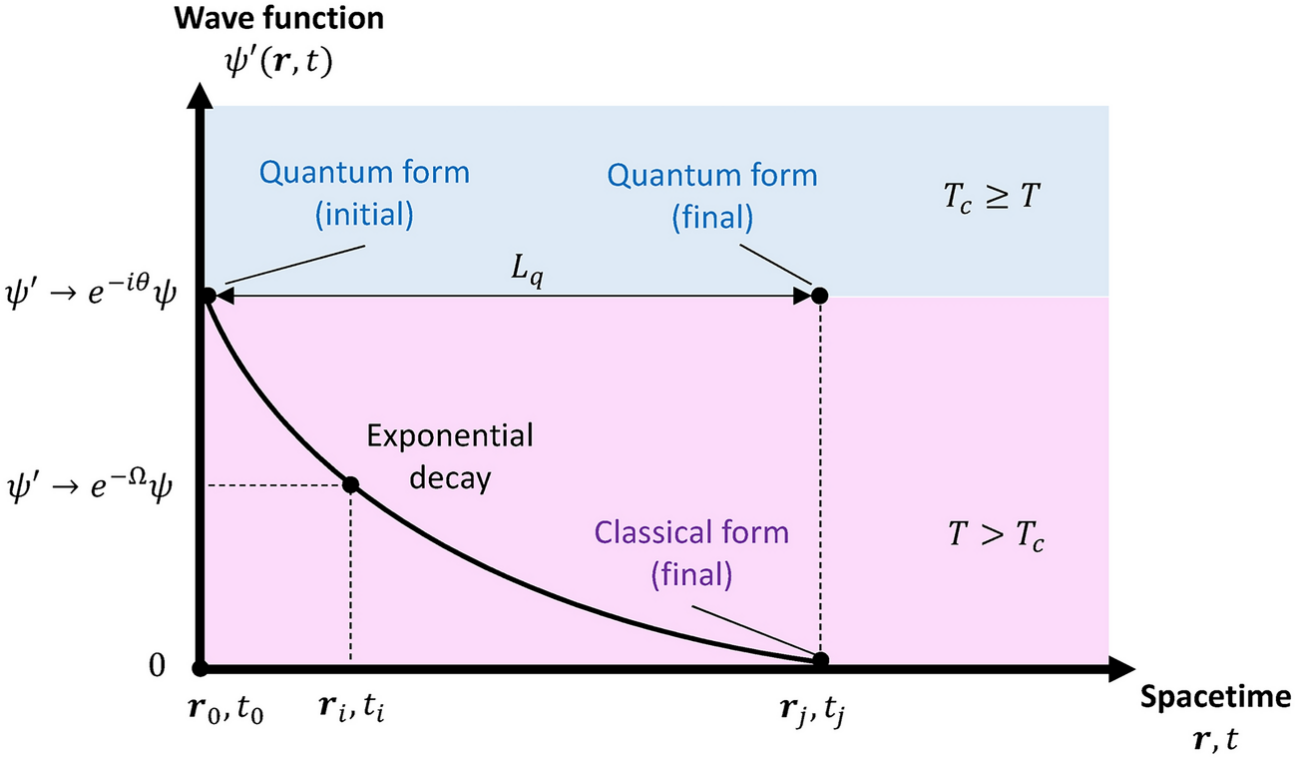
Transient from initial quantum form into final classical form via exponential process.

Within this dimension, the wave function decays exponentially, according to the logarithmic decrement rule. Therefore, for a certain duration, we observed that a portion of the system is transient from the quantum form (at the initial state) to the classical form (at the final state) via exponential decay on the spatial and temporal scales, as shown in Fig. 3.

Since the square modulus of the wave function ${\left|{\psi }^{\prime}\right|}^{2}$ is associated with the probability in quantum formulation, the associated quantum (wave-like) effect decays exponentially within the dimension $\left|{\varvec{r}}\right|\le {L}_{q}$. As a result, for a given temperature and initial condition, the distance reflects the degree of the quantum-to-classical transition in the thermodynamic system. This reveals that depending on the distance, the established field quantization and its associated wave-like behavior are only locally effective and vary with respect to the critical distance. Influence of quantum effect on the outside range $\left|{\varvec{r}}\right|>{L}_{q}$ becomes negligible and the system is governed by the classical formulation. Consequently, it interprets the transition of quantum formulation to classical formulation in finite spatial and temporal limit.

## Methods

### Heat transport and thermodynamics

As in the literature, the Fourier law of heat transport is usually given in the differential form of the temperature field as follows^2^:

60 $${\varvec{q}}=-\kappa \nabla T,$$

where $\kappa$ is the heat conductivity, and ${\varvec{q}}$ denotes the local heat flux, which is spatially dependent only. The above equation assumes that the local thermal equilibrium has been reached everywhere in the spatial domain. The energy balance of the element in local thermal equilibrium can be represented as

61 $${\partial }_{t}T+\frac{\kappa }{\rho {C}_{p}}\nabla T=0 ,$$

where $\rho$ is the mass density, ${C}_{p}$ is the specific heat capacity, and the heat convection and radiation are neglected. The internal energy of the system $U$ is given by the temperature and heat charge as follows:

62 $$U(T)=\iiint \rho {C}_{p}\Delta Td{{\varvec{r}}}^{3} ,$$

The enthalpy of continuous medium can be represented by:

63 $$\Delta H=\Delta U(T)+W=\Delta U(T)+\iiint \Delta Pd{{\varvec{r}}}^{3},$$

In the reversible process, the entropy of the system is defined as the quotient between the total energy and temperature as follows:

64 $$\Delta S=\frac{\partial H}{\partial T}=\frac{\partial }{\partial T}\left[\Delta U\left(T\right)+\Delta W\left(P\right)\right] \Rightarrow \frac{\partial U(T)}{\partial T}$$

where $\Delta W$ is temperature-independent and thus this term is entropy-free.

### Electromagnetism and gauge symmetry

Gauge symmetry is a fundamental property of gauge field theory that provides a description of particles and fields in quantum mechanics^40^. For electromagnetism with the Lorenz gauge function $\Lambda$:

65 $$\nabla \cdot {\varvec{A}}+\frac{1}{{{\varvec{c}}}^{2}}{\partial }_{t}V=0 \Rightarrow {\nabla }^{2}\Lambda -\frac{1}{{{\varvec{c}}}^{2}}{\partial }_{t}^{2}\Lambda =0$$

and

66 $$\Delta V=-{\partial }_{t}\Lambda , \Delta {\varvec{A}}=\nabla\Lambda ,$$

where $\Delta V$ denotes the change in the scalar potential and $\Delta {\varvec{A}}$ denotes the change in the vector potentials in different configurations. The Schrödinger equation remains unaltered when the following transformations of the wave functions from the old to new configurations are performed using the phase function ($\psi \to \psi ^{\prime}$):

67 $${\psi }^{\prime}=\lambda \circ \psi ={e}^{-i{\theta }_{\lambda }\left({\varvec{r}},t\right)}\psi ; \lambda \left({\varvec{r}},t\right)={e}^{-i{\theta }_{\lambda }\left({\varvec{r}},t\right)}=\frac{q}{\hslash }\Lambda \left({\varvec{r}},t\right),$$

where $\lambda$ denotes the complex phase function, which is space and time dependent. From the above relationship, the scalar and vector potentials can be derived from the gauge function in Eq. (60):

68 $$\varphi =-{\partial }_{t}\Lambda =\frac{\hslash }{q}\omega \lambda \left({\varvec{r}},t\right) ;{\varvec{A}}=\nabla\Lambda =\frac{\hslash }{q}{\varvec{k}}\lambda \left({\varvec{r}},t\right).$$

## Data Availability

All data generated or analyzed during this study are included in this published article.
